# Advances in Aptamer-Based Biomarker Discovery

**DOI:** 10.3389/fcell.2021.659760

**Published:** 2021-03-16

**Authors:** Jie Huang, Xinxin Chen, Xuekun Fu, Zheng Li, Yuhong Huang, Chao Liang

**Affiliations:** Department of Biology, Southern University of Science and Technology, Shenzhen, China

**Keywords:** aptamer, biomarker discovery, SOMAScan, CELL-SELEX, human diseases

## Abstract

The discovery and identification of biomarkers promote the rational and fast development of medical diagnosis and therapeutics. Clinically, the application of ideal biomarkers still is limited due to the suboptimal technology in biomarker discovery. Aptamers are single-stranded deoxyribonucleic acid or ribonucleic acid molecules and can selectively bind to varied targets with high affinity and specificity. Compared with antibody, aptamers have desirable advantages, such as flexible design, easy synthesis and convenient modification with different functional groups. Currently, different aptamer-based technologies have been developed to facilitate biomarker discovery, especially CELL-SELEX and SOMAScan technology. CELL-SELEX technology is mainly used to identify cell membrane surface biomarkers of various cells. SOMAScan technology is an unbiased biomarker detection method that can analyze numerous and even thousands of proteins in complex biological samples at the same time. It has now become a large-scale multi-protein biomarker discovery platform. In this review, we introduce the aptamer-based biomarker discovery technologies, and summarize and highlight the discovered emerging biomarkers recently in several diseases.

## Introduction

Biological markers, also named as biomarkers, are usually considered as indicators of specific biological conditions in normal and pathogenic processes, or possible pharmacologic responses to therapeutics (Aronson and Ferner, 2017). Biomarkers (such as proteins, hormones, enzymes) exist in patient’s blood, body fluids, cells, or tissues that are abnormally produced or whose expression is altered by changes in the gene expression of diseased cells during the occurrence and development of the disease. Therefore, biomarkers are of great significance for the characterization of specific physiological and pathological conditions in all living organisms. Biomarkers have become an increasingly important part of clinical medicine, as well as bench and bedside research. As for clinical application, biomarkers are mainly used for diagnosis, prognosis and risk prediction (Califf, 2018). For instance, blood glucose or HbA1c can be used as diagnostic biomarkers for type 2 diabetes (Sherwani et al., 2016). Natriuretic peptide and cardiac troponin are widely used as biomarkers for the diagnosis and prognosis of heart failure and acute myocardial infarction (Lyngbakken et al., 2019). Serum prostate specific antigen is widely used in the diagnosis of prostate cancer, the prognosis after treatment, the clinical efficacy of drugs and the monitoring of disease recurrence (Sanhueza and Kohli, 2018). Therefore, the exploitation and research of biomarkers are of great significance for early diagnosis and targeted therapy of many diseases.

Normally, mass spectrometry (MS) and antibody-based technology are employed to explore new biomarkers that are beneficial for diagnosing and managing diseases (Cifani and Kentsis, 2017; Kowalczyk et al., 2020). MS-based proteomics, a powerful technology for the study of biomarkers, can obtain highly accurate peptide mass and fragment spectrum from protein sequence-specific digestion products (Geyer et al., 2017). Using liquid chromatography-tandem MS (LC-MS/MS), researchers have found many biomarker candidates in lung cancer, chronic obstructive pulmonary disease and asthma, such as A1BG, LRG1, ERO1L, NARS, TGM2, S100A9 (Fujii et al., 2017). However, these technologies still have many unresolved challenges. The biggest one is the low sensitivity especially for the detection of analytes with concentration below 50 pg/mL. Besides, the intensive sample preparation process may cause false positive signals (Mahmud and Garrett, 2020). To solve these problems, researchers start to apply antibodies with high affinity for their targets to detect specific proteins. For example, enzyme-linked immunosorbent assay and enzyme immunoassay were established by connecting enzymes with antibody antigen, respectively (Yiling et al., 2018). In addition, planar arrays like single antibody arrays and sandwich antibody arrays have also been developed and used for a large-scale protein detection (Eickhoff and Malik, 2013). Antibody-based assays solve the low sensitivity problem of MS and can detect analytes in the sub-nM range, which make this technology become the most widely used method for protein detection currently. Unfortunately, these issues of cross reactivity, poor reproducibility, complexity and high cost are still unsolved. Apart from that, the early diagnosis of tumors and other major diseases is still an open question due to the lack of appropriate and specific biomarkers. Therefore, it is rather essential to develop desirable affinity reagents that allow researchers to explore proteins effectively and efficiently.

Aptamers are single-stranded deoxyribonucleic acid (ssDNA) or ribonucleic acid (RNA) molecules and can solve these problems above mentioned. Studies have shown that aptamers can target almost any proteins and have a dissociation constant at the level of picomolar to nanomolar (Cole and Lupták, 2019). Generally, aptamers fold into a specific tertiary structure and bind to targets such as proteins, metals, and molecules (Ellington and Szostak, 1990; Tuerk and Gold, 1990; Cole and Lupták, 2019), and aptamers could be used as carriers of therapy for accurate delivery of therapeutic drugs, such as chemotherapy drugs, small interfering RNAs, microRNAs, antisense oligos, or toxins, to the target cells but not to normal cells (Soldevilla et al., 2018). These aptamer-drug conjugates (ApDCs) have gradually become effective drug delivery systems and attracted widespread attention (Xuan et al., 2018). Aptamers typically contain 25–100 nucleotides and can be produced through systematic evolution of ligands through exponential enrichment (SELEX) (Nimjee et al., 2017). By applying SELEX, aptamers can be selected against a wider range of molecular biomarkers that are difficult to generate antibodies against, including toxins and biomarkers with poor immunogenicity. So far, the SELEX technology have been employed in diverse applications including therapeutics, catalysis, and now proteomics (Gragoudas et al., 2004; Gold et al., 2010; Zhuo et al., 2017). Compared with traditional antibodies, aptamers have extra exceptional advantages, such as flexible design, low cost, easy synthesis, convenient modification with different functional groups and poor immunogenicity (Ali et al., 2019). Thus, aptamer-based technology can promote a wide range of diagnostic and therapeutic applications and the detection of new biomarkers.

Given that aptamers are attractive affinity reagents and have multiple advantages, an increasingly number of researches about aptamer-based biomarker discovery were carried out over the past decades. Up to now, several aptamers have been used for disease treatment and biomarker detection. For example, the US Food and Drug Administration approved the VEGF165 inhibitory aptamer pegaptanib (Macugen) in 2004 for the treatment of age-related macular degeneration (Ng et al., 2006). Protein tyrosine kinase 7 (PTK7) identified by aptamer Sgc8 in leukemia cell possibly makes aptamer Sgc8 become a diagnostic biomarker in the future (Shangguan et al., 2008). Meanwhile, a variety of screening technologies about aptamer-based biomarker discovery are improved to identify biomarkers for disease diagnosis, management and prognosis, such as CELL-SELEX and SOMAScan. In this review, the following sections will introduce technologies of aptamer-based biomarker discovery and biomarkers identified by these technologies in diseases, such as cancer, neurodegenerative disease, cardiovascular disease, and other diseases (Table 1).

**TABLE 1:** Emerging biomarkers based on aptamer in various diseases.

Diseases	Biomarkers	Methods	References
**Cancer**			
Nasopharyngeal carcinoma	CD109	CELL-SELEX	Jia et al., 2016
Ovarian cancer	STIP1	CELL-SELEX	Van Simaeys et al., 2014
	APOA1, CGB, FSHB, IL-6, MMP7	SOMAScan	Graumann et al., 2019
	BDNF, MDC, PAI1, PDGF etc.	SOMAScan	Mysona et al., 2019
Pancreatic cancer	ALPPL-2	CELL-SELEX	Dua et al., 2013
	CypB	CELL-SELEX	Ray et al., 2012
Glioblastoma	Tenascin C	CELL-SELEX	Daniels et al., 2003
NSCLC	Cadherin-1, CD30 Ligand, Endostatin, PRKCI, RGM-C, SCF Sr, sL-Selectin, YES	SOMAScan	Ostroff et al., 2010
SCLC	HDLBP	CELL-SELEX	Zhou et al., 2019
Hepatocellular carcinoma	ApoA 1	−	Mustafa et al., 2015
Tumor endothelial cells	Troponin T	CELL-SELEX	Ara et al., 2014
Leukemia	PTK7	CELL-SELEX	Shangguan et al., 2008
**Neurodegeneration-related diseases**			
Parkinson’s disease	BSP, OMD, ACY1, GHR	SOMAScan	Posavi et al., 2019
Multiple sclerosis	KLKB1, ApoE4, DKK3, C6, S100A9 etc.	SOMAScan	Welton et al., 2017
	MMP7, SERPINA3, GZMA, CLIC1, DSG2, TNFRSF25	SOMAScan	Masvekar et al., 2019
**Cardiovascular diseases**			
MI	FABP, SDF-1	SOMAScan	Ngo et al., 2016
	Cysteine, CSRP3, ATP5J	SOMAScan	Jacob et al., 2018
Heart failure	ANG-2, THBS-2	SOMAScan	Wells et al., 2019
	NT-proBNP, THBS-2, MBL, EGFR, GDF-11/8, Hemojuvelin	SOMAScan	Nayor et al., 2020
	Angiopoietin-2, THBS-2, LTBP-4, FSTL3	SOMAScan	Chan et al., 2020
Cardiac hypertrophy	GDF11	SOMAScan	Loffredo et al., 2013
Acquired aplastic anemia	CCL17, DKK1, HGF, SELL	SOMAScan	Giudice et al., 2018
**Other diseases**			
Pulmonary tuberculosis	CF-V, XPNPEP1, PSME1, IL-11 Rα, HSP70, Galectin-8,	SOMAScan	Nahid et al., 2014
	α2- Antiplasmin, ECM1, YES, IGFBP-1, CATZ, BGN, LYNB, and IL-7; NECL, KLRK1		
	TSP4, TIMP-2, SEPR, MRC-2, Antithrombin III, SAA, CRP, NPS-PLA2, LEAP-1, LBP	SOMAScan	De Groote et al., 2013
Rheumatoid arthritis	IL-16	SOMAScan	Murota et al., 2016
CKD	cystatin C, b2-MG, CFD, TNF sR-I etc.	SOMAScan	Gold et al., 2010
Acute kidney injury	FGF23, tPA, MMP8, suPAR	SOMAScan	Yu et al., 2018
DMD	ATL1, Myoglobin	SOMAScan	Hathout et al., 2015
	CYCS, TPI1, and THBS4	SOMAScan	Coenen-Stass et al., 2015
Preterm birth	CFB, CFH, CF-IX, CF-IX ab	SOMAScan	Lynch et al., 2016
Metabolic syndrome	HGF, RTK FLT3, BSP2, GKRP, ESM-1	SOMAScan	Patel et al., 2018
IPF	Glycoproteins THBS1, vWF, CCL17, BPI; ROBO2, Spo-1, plgR, ICAM5	SOMAScan	Todd et al., 2019
UTI	Bcl protein, CXCL 6, CXCL 13, CTSS, HSPA1A, MAPK, HPV18-E7, TAGLN	SOMAScan	Dong et al., 2020

*NSCLC, Non-small cell lung cancer; SCLC, Small cell lung cancer; MI, Myocardial infarction; DMD, Duchenne muscular dystrophy; IPF, Idiopathic pulmonary fibrosis; CKD, Chronic kidney disease; CD109, Cluster of differentiation 109; STIP1, Stress-induced phosphoprotein 1; ApoA1, Apolipoprotein A1; CGB, Cytoglobin; FSHB, Follicle Stimulating Hormone b; IL-6, Interleukin 6; MMP7, Matrix metalloproteinase 7; BDNF, Brain Derived Neurotrophic Factor; MDC, Macrophage Derived Chemokine; PAI1, Plasminogen activator inhibitor-1; PDGF, Platele tderived growth factors; ALPPL-2, Alkaline phosphatase placenta-like 2; CypB, Cyclophilin B; RGM-C, Repulsive guide molecule C; HDLBP, High density lipo-binding protein; PTK7, Protein tyrosine kinase; SAA, Serum amyloid A; XPNPEP1, X-Prolyl Aminopeptidase; PSME1, Proteasome Activator Subunit 1; IL-11, Interleukin-11; HSP 70, Heat shock protein 70; ECM 1, Extracellular matrix 1; IGFBP-1, Insulin-like growth factor-binding protein-1; BGN, Bilycan; NECL, Nectin-like protein; KLRK1, Killer Cell Lectin Like Receptor K1; TSP4, Human thrombospondin 4; TIMP-2, Tissue inhibitor of metalloproteinase-2; MRC-2, Mannose receptor C 2; CRP, C-reactive protein; LBP, Lipopolysaccharide binding protein; MCP, Major capsid protein; BSP, Bone sialoprotein; OMD, Osteomodulin; ACY1, Aminoacylase-1; GHR, Growth hormone receptor; KLKB1, Kallikrein B1; DKK3, Dickkopf-3; GZMA, Granzyme A; CLIC1, Chloride intracellular channel 1; SERPINA3, Serpin peptidase inhibitor A3; DSG 2, Desmoglein 2; TNFRSF25, Tumor necrosis factor receptor superfamily member 25; FABP, Fatty acid binding protein; SDF-1, Stromal derived factor-1; CSRP 3, Cysteine and glycine-rich protein 3; ATP5J, ATP synthase F0 subunit component; AGN-2, Angiopoietin-2; THBS-2, Thrombospondin-2; NT-proBNP, N-terminal proB-type natriuretic peptide; MBL, Mannose-binding lectin; EGFR, Epidermal growth factor receptor; GDF-11/8, Growth differentiation factor-11/8; LTBP-4, Latent transforming growth factor-β binding protein-4; FSTL3, Follistatin-related protein-3; GDF11, Growth Differentiation Factor 11; DKK1, Dickkopf-1; HGF: Hepatocyte growth factor; SELL, Selectin leukocyte L; CF-V, Coagulation Factor V; FGF-23, Fibroblast growth factor-23; Tpa, Tissue plasminogen activator; MMP8, Matrix metalloproteinase-8; suPAR, Soluble urokinase plasminogen activator receptor; ATL1, Alanine aminotransferase 1; CYCS, cytochrome c; TPI1, Triosephosphate isomerase 1; THBS4, Thrombospondin 4; CFB, CFH, Complement factors B and H; CF-IX, the coagulation factors IX; CF-IX ab, the coagulation factors IX antibody; RTK, receptor tyrosine kinase; FLT3, fms related tyrosine kinase 3; BSP2, Bone sialoprotein 2; GKRP, Glucokinase regulatory protein; ESM-1, Endothelial cell-specific molecule 1; THBS1, Thrombospondin 1; Vwf, Von willebrand factor; CCL17, C-C modify chemokine ligand 17; BPI, Bactericidal permeability-increasing protein; ROBO2, Roundabout homolog-2; Spo-1, Spondin-1; plgR, Polymeric immunoglobulin receptor; ICAM5, Intercellular adhesion molecule 5; b2-MG, b2-microglobulin; CFD, Complement factor D; Bcl, B-cell lymphoma; CXCL 6, CXC motif chemokine 6; CXCL 13, CXC motif chemokine 13; CTSS, Cathepsin S; HSPA1A, Heat shock kDa protein 1A; MAPK, Mitogen activated protein kinase; HPV18-E7, Human papilloma virus type 18 protein E7; TAGLN, Transgelin.*

## Advanced Technologies for Discovering Biomarkers Based on Aptamers

For traditional SELEX technologies, it is quite difficult to obtain proteins with high enough purity from various and complex expression systems *in vitro*, especially for intracellular proteins and transmembrane proteins. Therefore, researchers have been studying on developing new technologies for aptamer selection, such as microfluidic SELESX, *in silico* SELEX, bead-based SELEX, *in vivo* SELEX, and so on (Bayat et al., 2018). In order to use aptamers to detect biomarkers, related technologies have been greatly developed in recent years, and the CELL-SELEX technology and SOMAScan technology have been widely adopted.

### CELL-SELEX Technology

**FIGURE 1:**
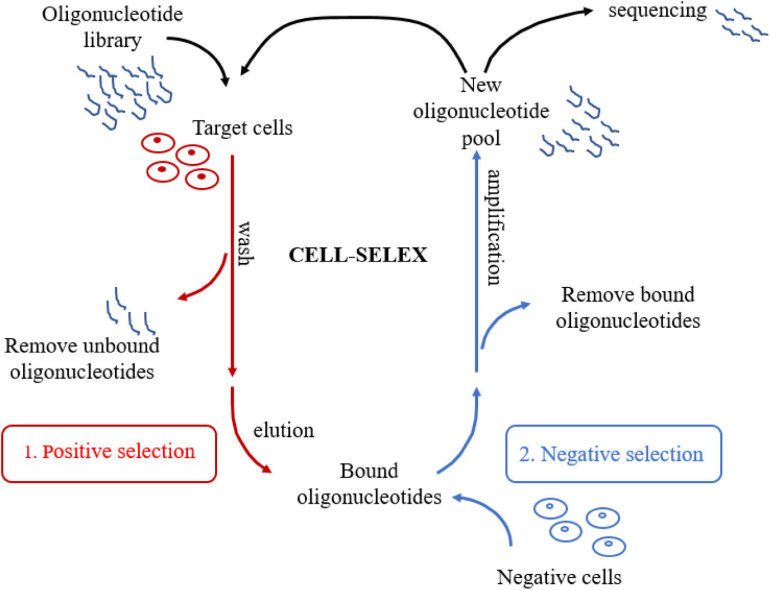
Schematic representation of CELL-SELEX. The process involves repeated cycles of: **(1)** Target cells are firstly incubated with the initial oligonucleotide library for positive selection; **(2)** Bound oligonucleotides are incubated with negative cells for negative selection, then unbound oligonucleotides were amplified to generate a new library of oligonucleotides for the next selection round. After about 20 cycles, several aptamers can be selected. Finally, Candidate aptamers are selected by cloning and sequencing the aptamer pool.

Unlike other SELEX technologies, CELL-SELEX directly screens aptamers for the whole cell. Molecular targets on the cell membrane surface are in a native folded state, which further improves the *in situ* efficiency of the selected aptamers (Sefah et al., 2010). There are several significant advantages of CELL-SELEX. In the first place, this technology is able to select aptamers that can distinguish normal cells from diseased cells without knowing the difference between cells in advance. Moreover, CELL-SELEX eliminates the problem that identified aptamers can only bind to purified proteins but not proteins in their native state. Finally, CELL-SELEX also can be applied to develop new biomarkers in disease cells (Chen et al., 2016). As for the procedure of CELL-SELEX (Figure 1), the target cells are firstly incubated with the initial ssDNA or RNA library for positive selection. Heat treatment eluted bound sequences after removing the unbound oligonucleotides. Next, the eluted oligonucleotides are incubated with control cells for negative selection. Only unbound sequences that specifically recognize target cells were kept after eliminating the sequences binding to cell membrane surface proteins shared by two cell lines. Consequently, the oligonucleotides that are not bound during the negative selection process are amplified by PCR to generate a new library of oligonucleotides for the next selection round. A flow cytometer is used to assess the progress of the screening. After about 20 cycles of selection, several aptamers can be developed. The next stage is to clone and sequence the corresponding aptamer pool. Finally, candidate aptamers are selected. These selected aptamers are able to distinguish certain cell lines from other cells, such as cancer cells, tumor cells or other disease cells, and even different subtypes of cells (Sefah et al., 2010).

In 1998, Morris and Jensen used CELL-SELEX for the first time to screen aptamers by using human red blood cell membrane as a complex mixed target. This technology provides an *in vitro* procedure that can screen aptamers with high affinity against complex mixture targets, such as lymphocytic leukemia, liver cancer, and so on (Morris et al., 1998). Based on previous works of predecessors, Tan’s group established a systematic CELL-SELEX procedure to explore cell membrane biomarkers (Shangguan et al., 2006). In 2008, Berezovski et al. (2008) proposed a technique for discovering biomarkers, namely aptamer-facilitated biomarker discovery (AptaBiD). This technology was based on multiple rounds of aptamer screening and amplification, and could detect small differences in molecular targets between two cell populations with high sensitivity (Berezovski et al., 2008). Actually, the AptaBiD technology also aimed at the whole cell to discover biomarkers. In the past two decades, researchers have identified a range of aptamers based on CELL-SELEX for various living cells and other complex systems, especially tumor and cancer cells (Fang and Tan, 2010).

### SOMAScan Technology

**FIGURE 2:**
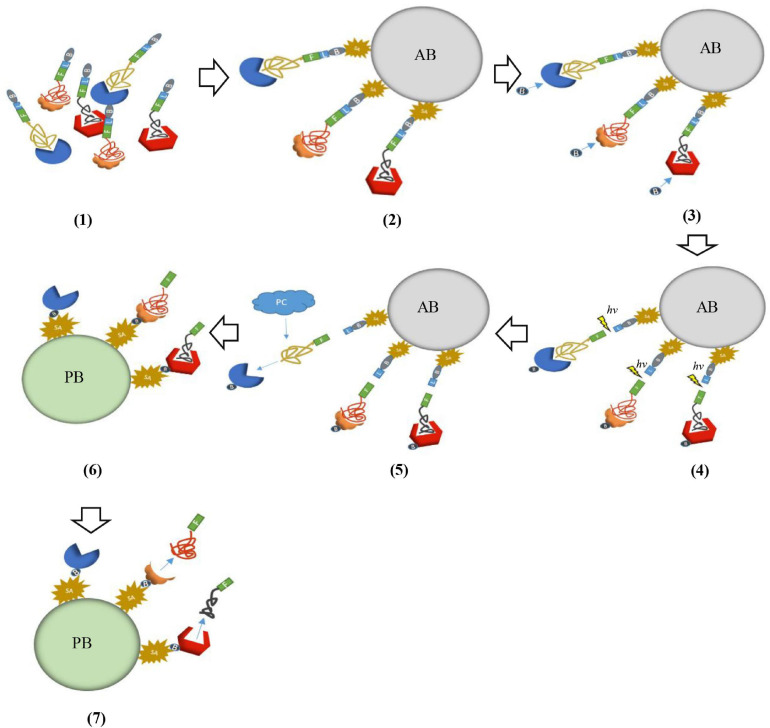
Schematic representation of SOMAScan. **(1)** SOMAmers are mixed with the target sample forming SOMAmer-protein complexes. These complexes are tagged with biotin (B) and fluorescent label (F). **(2)** These complexes are captured onto avidin bead (AB) with streptavidin (SA). **(3)** The captured proteins were labeled with biotin. **(4)** The complexes were released from beads through ultraviolet light (*hv*). **(5)** Polyanionic competitors (PC) were added to promote the dissociation between proteins and non-specific SOMAmers. **(6)** Bound complexes are captured onto primer beads (PB). **(7)** Captured complexes are dissociated with 20 mM NaOH.

SOMAScan is a multiplexed proteomic platform based on slow off-rate modified aptamers (SOMAmers). Compared with traditional aptamers, the biggest advantage of SOMAmers is that the binding force between the oligonucleotides in the nucleotide library and the target protein is significantly enhanced because of chemical modification. Due to the great advantage in dissociation constants of SOMAmers, a continuous two-step capture strategy is introduced in SOMAScan assay to minimize non-specific binding and avoid cross reaction. Therefore, this technology can avoid the impediment of low sensitivity of MS and failure of multiplexing of immunoassay methods in the detection of cancer-related biomarkers (Gold et al., 2010). In brief, the first step is that SOMAmers are labeled with biotin by UV-cleavable linkers, they are firstly mixed with the sample and incubated to reach equilibrium. The reaction solution is incubated with streptavidin beads and unbound proteins are washed away. Proteins combined with SOMAmers are labeled with NHS-biotin and fluorescent label. The UV-cleavable linkers are cleaved by ultraviolet light, and these complexes are eluted. The second step is that using monomeric avidin beads captures these complexes labeled by the biotin. In this process, high-concentration polyanions (such as dextran sulfate) can act as common competitors to remove non-specific binding SOMAmers. The next step is that SOMAmer-protein complexes are captured by the complementary oligonucleotide primers of SOMAmers. Then all remaining unbound proteins are washed. Finally, these captured complexes are separated by NaOH and target proteins are eluted (Figure 2). Target proteins are analyzed by denaturing polyacrylamide gel electrophoresis (PAGE) and MS (Gold et al., 2010).

In 2010, Gold et al. (2010) invented SOMAScan technology and successfully applied it to screen 58 potential biomarkers through analyzing plasma of patients with chronic kidney disease, such as b2-MG, pleotrophin, CFD, Cystatin C and so on (Gold et al., 2010). After that, due to extraordinary advantages of SOMAScan technology, SOMAmer-based proteomics assays have been widely used in the development of large-scale disease biomarkers based on complex biological samples in various diseases, including malignant pleural mesothelioma (MM) (Ostroff et al., 2012), non-small cell lung cancer (NSCLC) (Ostroff et al., 2010), and myocardial injury (Ngo et al., 2016). In 2012, Baird et al. (2012) used cerebrospinal fluid (CSF) samples to identify 248 Parkinson’s disease potential biomarkers by SOMAScan assay, such as myoglobin, siglec.3, TNFsR.II, and so on. Furthermore, Webber’s team used the SOMAScan platform to discover 300 protein biomarkers previously unknown that associated with prostate cancer exosomes, like NOTCH-3, L1CAM, RAC1, ADAM9, and so on (Webber et al., 2014). These studies showed that SOMAScan technology is becoming a powerful universal platform for quickly and efficiently discovering multiple disease-related biomarkers from various complex biological samples.

## Biomarker Discovery Based on Aptamers in Various Diseases

For a long time, the diagnosis, management, and prognosis of various diseases have been perplexing both people and doctors. Since many diagnosed patients are generally at a terminal stage, many diseases are difficult to be cured. Meanwhile, some traditional therapies have great side effects because they cannot effectively distinguish diseased cells from normal cells, which increases the physical and psychological burden of patients. Therefore, cell-based diagnosis and targeted therapy are of great significance to improve the survival rate and alleviate the suffering of patients. Precision medicine needs to identify more reliable disease biomarkers. Biomarker discovery based on aptamers in various diseases has always been the focus of researchers.

### Cancers

T-cell acute lymphoblastic leukemia (T-ALL) is an aggressive malignant tumor with a deeply poor prognosis (Teachey and O’Connor, 2020). As early as 2008, Shangguan et al. (2008) used CELL-SELEX to detect a variety of biomarkers of a series of leukemia cell lines and discovered a transmembrane receptor tyrosine kinase-like molecule, protein tyrosine kinase 7 (PTK7), which highly expressed in these cell lines. Finally, PTK7 was identified as a potential biomarker for T-ALL by MS (Shangguan et al., 2008).

Nasopharyngeal carcinoma (NPC) is a head and neck squamous cell carcinoma with a high local recurrence or metastasis rate (Xiao and Chen, 2019). Using CELL-SELEX technology, Jia et al. (2016) screened four aptamers (S3, S5, S12, and S27) that could distinguish the molecular difference between NPC cells and normal nasopharyngeal (NP) cells. Meanwhile, the membrane protein CD109 was identified as the target protein of S3 aptamer. Further, they proved that CD109 was also expressed in NPC stem-like cells (CSCs). Their work showed that CD109 could be used as a potential biomarker of NPC for early cancer diagnosis and targeted therapy. Several studies had also revealed that CD109 was overexpressed in many types of tumors, such as human lower-grade glioma (Shiraki et al., 2017), myxofibrosarcoma (Emori et al., 2015), epithelial sarcomas (Emori et al., 2013), lung squamous cell carcinoma (Sato et al., 2007), cervical cancer (Zhang et al., 2005), cutaneous squamous cell carcinoma (Dong et al., 2014). Therefore, aptamer S3 was used to treat not only NPC, but also other cancers with abnormal CD109 expression (Jia et al., 2016).

Ovarian cancer (OC) is a disease with multiple subtypes (Kossaï et al., 2018). Van Simaeys et al. (2014) tested the target of the aptamer TOV6 obtained by CELL-SELEX for OC previously. Stress-induced phosphoprotein 1 (STIP1) was identified as the target of TOV6. Graumann et al. (2019) used the antibody-based proximity extension assay (PEA) platform to compare and analyze the plasma of patients with OC and other non-malignant gynecological diseases. One hundred seventy six highly expressed proteins were identified in OC patients. By using SOMAScan, they verified that APOA1, CGB, FSHB, IL-6, MMP7 could be used as potential diagnostic biomarkers in OC (Graumann et al., 2019). In another study, Mysona et al. (2019) used SOMAScan to continuously exam 1129 proteins in the blood of patients with OC. Twenty six proteins verified by Luminex test had a positive correlation with OC recurrence, like BDNF, MDC, PAI1, PDGF, and so on (Mysona et al., 2019). Their works are meaningful for predicting the prognosis of OC patients after treatment remission, and are expected to provide early intervention and consolidation treatment for patients who may relapse.

Pancreatic cancer (PC) is a solid malignancy with extremely high mortality (Gupta et al., 2017). Using the CELL-SELEX, Dua et al. (2013) screened an aptamer (SQ-2) that specifically recognized PC cells. Then ALPPL-2 was identified as the biomarker of SQ-2. Their research found that ALPPL-2 was abnormally expressed in many PC cell lines and could enter the bloodstream as a kind of cell secretion. Therefore, ALPPL-2 in serum and membrane showed its potential for PC diagnosis (Dua et al., 2013). Through the same technology, Ray et al. (2012) screened an M9-5 aptamer that could distinguish PC from non-cancerous pancreatic cell lines. CypB was identified and verified as the biomarker of M9-5 by MS. CypB could be also used as a promising biomarker for early detection of PC (Ray et al., 2012).

Glioblastoma is considered to be an inherent brain tumor originating from glial stem cells or progenitor cells (Le Rhun et al., 2019). Daniels et al. (2003) successfully used U251 cell line as samples and screened the aptamer GBI-10 through CELL-SELEX. The molecular biomarker tenascin C of aptamer GBI-10 on U251 cells was identified and verified by MS and had a highly expression in the cell line (Daniels et al., 2003). This was the first practice of using the CELL-SELEX strategy to select aptamers and identify cellular proteins as a tumor biomarker.

Lung cancer (LC) is a complex disease, composed of a variety of histological and molecular types with clinical significance (Rodriguez-Canales et al., 2016). To explore potential biomarkers of NSCLC, Gold et al. (2010) used SOMAScan to measure 813 serum proteins. They found 44 potential biomarkers and established a 12-protein group (cadherin-1, endostatin, HSP 90α, LRIG3, CD30 ligand, MIP-4, pleiotrophin, PRKCI, RGM-C, SCF sR, sL-Selectin, YES) to diagnose NSCLC (Ostroff et al., 2010). Zhou et al. (2019) screened aptamer C1, C3, C7, and C12 for small cell lung cancer (SCLC) through the CELL-SELEX, and high density lipo-binding protein (HDLBP) was determined as their biomarkers. Their result showed that HDLBP was overexpressed in NSLC tissues and promoted the proliferation of SCLC cells by promoting the G1/S transition of the cell cycle. The development of HDLBP-based NSLC target therapy could be helpful for SCLC (Zhou et al., 2019).

Angiogenesis is very important for tumor formation, and endothelial cells (EC) are induced in the process of angiogenesis. Thus, the development of tumor EC biomarkers is significant for the early diagnosis of tumors. Ara et al. (2014) used CELL-SELEX to develop a high-affinity DNA aptamer AraHH001. They identified a new biomarker, namely troponin T, as the target of AraHH001 on primary cultured mouse tumor EC. They proved that troponin T was expressed in tumor EC, but not normal skin EC. As a result, troponin T was possibly a new tumor EC biomarker, which had a great potential in predicting the development of tumor metastasis and anti-angiogenic therapy (Ara et al., 2014).

Hepatitis C virus (HCV) is closely related to the occurrence of hepatocellular carcinoma (HCC), and the disease process from viral infection to HC is usually accompanied by changes in liver tissue and peripheral blood proteins (Mustafa et al., 2013). Mustafa et al. (2013) used aptamer-based fractionation technology (ProteoMiner) to reduce the complexity of serum. They found that compared with HCV patients, the level of ApoA1 in HCC patient was reduced. They totally found 24 proteins are differentially expressed between HCC and HCV patients, and ApoA1 could be a candidate serum biomarker for early HCC detection. It is meaningful for the early diagnosis of HCC developed from HCV virus infection (Mustafa et al., 2013).

### Neurodegeneration-Related Diseases

Parkinson’s disease (PD) is a common progressive neurodegenerative disease characterized by tremor and slow movement (Hayes, 2019). Posavi et al. (2019) used SOMAScan to detect 1,129 proteins in the serum of PD patients and normal people. They found that compared with people without PD, the levels of four proteins in the serum of PD patients showed the stable difference, namely BSP, OMD, ACY1, and GHR. They also compared the change in serum protein levels in PD patients longitudinally and showed that these proteins could be used to predict disease progression (Posavi et al., 2019).

Multiple sclerosis is a chronic central nervous system inflammatory disease of autoimmune etiology (Yamout and Alroughani, 2018). Welton et al. (2017) used SOMAScan to detect the CSF vesicles of patients with MS and found 50 proteins were particularly abundant in MS vesicles, such as KLKB1, ApoE4, DKK3, C6, S100A9 and so on. These proteins could be involved in the complement pathway, coagulation and wound healing. Their results showed that a part of the vesicle-related proteins of the CSF was meaningful for the discovery of neurological disease biomarkers and potential therapeutic targets (Welton et al., 2017). Masvekar et al. (2019) collected the CSF of 431 patients with neuroimmune diseases and healthy volunteers. Then taking the SOMAScan detect and analyze more than 1,000 proteins. They found that astrocyte cluster 8 and microglial cluster such as MMP7, SERPINA3, GZMA, CLIC1, DSG2, and TNFRSF25 were elevated in multiple sclerosis patients, and the elevation of these biomarkers was correlated with the severity of multiple sclerosis (Masvekar et al., 2019).

### Cardiovascular Diseases

Myocardial infarction (MI) is the leading cause of attributable postoperative death (Sessler and Khanna, 2018). Ngo et al. (2016) applied SOMAScan to measure 1129 proteins in “planned” myocardial injury (PMI). FABP and SDF-1 were identified as biomarker candidates in the peripheral vein blood. Jacob et al. (2018) adopted the same technology to assay 4,783 proteins in serum from patients with spontaneous MI and at-risk controls. They identified a number of novel biomarkers that were not previously found in the peripheral blood, or were as functional roles related to myocardial injury. In this study, the cardiac LIM protein cysteine and CSRP3 were thought to mediate stress responses and cardiac mechanotransduction while the mitochondrial ATP5J was a vasoactive peptide upon its release from cells (Jacob et al., 2018).

Heart failure (HF) presents a multifactorial, systemic disease and is considered as an epidemic disease in the modern world (Tanai and Frantz, 2015). A related HF study was performed by Wells et al. (2019) they retrieved plasma samples from patients with and without heart failure. They analyzed and screened these specimens by a SOMAScan assay. 9 candidate biomarkers were identified and two biomarkers, AGN-2 and THBS-2 were highly associated with HF (Wells et al., 2019). Recently, Nayor et al. (2020) evaluated 1895 Framingham Heart Study participants who underwent proteomic profiling and echocardiography. Using SOMAScan, they found 17 proteins associated with echocardiographic traits, and 8 of these proteins had pQTLs that associated with echocardiographic traits in EchoGen. In parallel, 3 proteins, NT-proBNP, THBS-2, MBL were associated with higher risk of HF. In addition, 3 proteins, EGFR, GDF11/8, and hemojuvelin were associated with lower risk (Nayor et al., 2020). Chan et al. (2020) found that 4 newly emergent biomarkers (AGN-2, THBS-2, LTBP-4, and FSTL3) were consistently associated with the development of post–MI HF.

Cardiac hypertrophy (CH) is a compensatory thickening of the heart caused by increased cardiac load (Nakamura and Sadoshima, 2018). In a study of Loffredo et al. (2013) they used a heterochronic parabiosis model that the blood circulation of a younger mouse was surgically joined with that of an older mouse. Through exposing to the circulation of a young mouse, CH declined dramatically after 4 weeks in the old mouse. Moreover, they identified the TGF-β superfamily member GDF11 by SOMAScan and the protein as a circulating factor was reduced with age. Treatment of restoring GDF11 to old mice suggested the effects of parabiosis and reversed age-related hypertrophy, which revealed a novel therapeutic chance for cardiac aging (Loffredo et al., 2013).

Acquired aplastic anemia (AA) is a kind of acquired bone marrow (BM) hematopoietic failure syndrome characterized by pancytopenia and BM hypocellularity (Young, 2013). In Giudice’s study, to identify new protein biomarkers in serum and plasma for diagnosis of AA and response to immunosuppressive therapies (IST), SOMAScan was applied to screen 1,141 serum proteins from 28 AA patients before and after therapy, 1,317 plasma proteins from 7 severe AA patients treated with standard IST and a thrombopoietin receptor agonist. 19 and 28 novel proteins from serum and plasma, respectively were identified as possible candidate diagnostic and prognostic biomarkers. After applying a custom immunobead-based multiplex assay, four biomarkers contained DKK1, HGF, CCL17, SELL, and HGF were verified and used for AA’s diagnosis and the long-term response to IST (Giudice et al., 2018).

### Other Diseases

Pulmonary tuberculosis (PTB) is an airborne disease caused by mycobacterium TB. It can affect the lung and cause severe cough, fever and chest pain (Fogel, 2015). To find new biomarkers in PTB, Nahid et al. (2014) used SOMAScan to compare and analyze serum samples collected from 39 PTB patients at baseline and 8 weeks after treatment. They identified 15 proteins associated with 8 week culture status that showed a significantly difference in PTB, including coagulation factor V, SAA, XPNPEP1, PSME1, IL-11 Rα, HSP70, galectin-8, α2-antiplasmin, ECM1, YES, IGFBP-1, CATZ, BGN, LYNB, and IL-7. In addition, some biomarkers, such as nectin and KLRK1, showed different changes between responder and slow-responder, or were related to transformation culture time (Nahid et al., 2014). De Groote et al. (2013) also found the expression of TSP4, TIMP-2, SEPR, MRC-2, antithrombin III, SAA, CRP, NPS-PLA2, LEAP-1, and LBP significantly changed between baseline and 8 weeks of therapy.

Rheumatoid arthritis (RA) is a chronic systemic autoimmune disease characterized by synovitis of the joints (Murota et al., 2016). Murota et al. (2016) analyzed 1,128 serum proteins from patients with RA, primary Sjogren’s syndrome and healthy patients by using SOMAScan. IL-16 showed the highest correlation with MMP-3 for using methotrexate to naïve and inadequate response RA patients based on synovitis status. On the other hand, considering that fluctuation of IL-16 was greatly associated with respond clinically when methotrexate or biologics were employed to treat RA, IL-6 was seen as a stronger clinical index than C-reactive protein, MMP-3 or erythrocyte sedimentation rate (Murota et al., 2016).

Acute kidney injury (AKI) has a high mortality, serum biomarkers may be useful to understand the pathophysiological processes involved with AKI and injury severity (Yu et al., 2018). Yu et al. (2018) profiled 1305 proteins in each serum sample. Higher serum levels of FGF23, tPA, MMP8, and suPAR, were significantly associated with the higher mortality. In the future, these biomarker candidates are expected to become predicators of kidney diseases (Yu et al., 2018).

Duchenne muscular dystrophy (DMD) is a recessive disease related to the X chromosome, and it mainly affects boys (Yiu and Kornberg, 2015). Hathout et al. (2015) used SOMAScan to conduct a large-scale biomarker detection on serum samples from DMD patients and age-matched healthy volunteers. They obtained 44 biomarkers present in the blood, including ATL1, myoglobin and so on. Among the 44 markers, 24 were significantly increased, and 20 were dramatically decreased. Their findings revealed potential emerging diagnostic and therapeutic strategies for reducing DMD and provided new protein biomarkers for DMD (Hathout et al., 2015). Coenen-Stass et al. (2015) also used SOMAScan to analyze the serum proteome of wild-type, malnourished and PPMO-treated mice (PPMO referred to the peptide-phosphorodiamidate morpholino oligonucleotide), and discovered a variety of new protein biomarkers of DMD. They found that CYCS, TPI1, THBS4 in malnutrition serum were elevated, and when dystrophin recovered, these proteins could return to wild-type levels, so they may be used to monitor the effect of treatment in patients with DMD. In addition, the concentration of ADAMTS5 was also found to increase in the serum of DMD patients. However, ADAMTS5 also was increased in becker muscular dystrophy and facioscapulohumeral muscular dystrophy patients, so ADAMTS5 might not be a specific biomarker and indicator of muscle pathology (Coenen-Stass et al., 2015).

Preterm birth (PB) is defined as the birth occurring before 37 weeks of gestation. It is a serious obstetric problem, which is related to the high morbidity and mortality of newborns (Suff et al., 2019). In 2005, a study revealed the relationship between circulating protein and preterm delivery in early pregnancy by SOMAScan. To identify proteins associated with PB in plasma at 10–15 weeks of gestation and to determine which protein pathways are vitally associated with PB, Lynch et al. (2016) detected 1129 proteins in serum from 41 women who give birth prematurely and 88 women who deliver at full term without complications. They found the CFB, CFH, and CF-IX, CF-IX ab were the highest-ranking proteins for PB.

Metabolic syndrome is a cluster of abnormalities with body about metabolism, like high blood pressure, diabetes, obesity, and atherosclerosis. Water T2 existed in plasma and serum are known as early, global, and practical biomarkers for metabolic syndrome and its underlying abnormalities (Patel et al., 2018). Patel et al. (2018) analyzed plasma samples from metabolic syndrome patients by SOMAScan assay to measure the relative concentrations of 1310 proteins. Five new proteins including HGF, RTK, FLT3, BSP2, GKRP, and ESM-1 had a positive correlation with water T2 (Patel et al., 2018).

Idiopathic pulmonary fibrosis (IPF) is a chronic, progressive, fibrotic, interstitial disease, which limitedly occurs in the lungs. In 2019, Todd et al. (2019) collected plasma from patients with IPF and without lung diseases and analyzed 1305 proteins by taking SOMAScan. 551 proteins were identified and had a significant difference between IPF and without IPF. There existed the greatest difference in levels among these proteins, such as the THBS1 and Vwf and CCL-17 and BPI protein. Robo2, Spo-1, PIR, ICAM-5 were shown to be positively correlated with the treatment of all three severe diseases (nintedanib, pirfenidone, neither). These proteins discovered with the most significant difference may symbolize biomarker indicators and implicate related pathways for further exploration (Todd et al., 2019).

Urinary tract infection (UTI) is the most common bacterial infection disease. Traditional diagnostic methods based on urine culture are time-consuming and not sensitive enough. Improving the accuracy of UTI diagnosis can help reduce unnecessary antibiotic use. Dong et al. (2020) used the SOMAScan platform to detect biomarkers associated with urine. They found eight potential protein biomarkers to diagnosis UTI, such as Bcl protein, CXCL 6, CXCL 13, CTSS, HSPA1A, MAPK, HPV18-E7, and TAGLN (Dong et al., 2020).

## Conclusion

As discussed in this review, researchers have discovered numerous biomarkers based on aptamer to be predictive, diagnostic or prognostic tools in various diseases, such as nasopharyngeal carcinoma, pancreatic cancer, RA, and so on. These studies suggest that aptamer-based biomarkers discovery broadens the road and increases the sensitivity to explore novel biomarkers. There is also no denying that the predictive, diagnostic or prognostic effect of these potential and emerging biomarkers still need to be verified at lots of pre-clinic and clinic trials. Besides, it is worth noting that single biomarkers are difficult to diagnose disease and clarify mechanisms. Therefore, biomarker panels should be highly recommended, and combining with clinical indicators to improve the predictive power of diseases. Overall, in the future, these predictive, diagnostic or prognostic biomarkers discovered by aptamer-based technologies could be promising and play a significant role in practice.

The CELL-SELEX and SOMAScan technology are more popular than other technologies, especially SOMAScan. In spite of their popularity, both of them still have some limitations. For CELL-SELEX, the major technical challenge in biomarker discovery is the aptamer target separation and validation, in particular membrane protein molecular targets. For SOMAScan, biomarkers need to be further quantified using other methods such as enzyme linked immunosorbent assay, and SOMAmers are specific only to the proteins they select. These problems are gradually being solved due to the continuous advancements of research methods. Thus, aptamer-based biomarker discovery shows a promising future.

## Author Contributions

CL designed and supervised the manuscript. JH and XC consulted literatures and wrote the manuscript in equal contribution. XF, ZL, and YH proposed advice to the manuscript. All authors read the manuscript and agreed to publish it.

## Conflict of Interest

The authors declare that the research was conducted in the absence of any commercial or financial relationships that could be construed as a potential conflict of interest.
